# Nutritional assessment using subjective global assessment identifies energy malnutrition and predicts mortality in patients with liver cirrhosis

**DOI:** 10.1038/s41598-025-89803-6

**Published:** 2025-02-09

**Authors:** Takao Miwa, Tatsunori Hanai, Kayoko Nishimura, Sachiyo Hirata, Shinji Unome, Yuki Nakahata, Kenji Imai, Atsushi Suetsugu, Koji Takai, Masahito Shimizu

**Affiliations:** 1 Department of Gastroenterology/Internal Medicine, Graduate School of Medicine, Gifu University, 1-1 Yanagido, Gifu, 501-1194 Japan; 2 Center for Nutrition Support & Infection Control, Gifu University Hospital, Gifu, Japan; 3 Department of Gastroenterology, Asahi University Hospital, Gifu, Japan; 4 Division for Regional Cancer Control, Graduate School of Medicine, Gifu University, Gifu, Japan

**Keywords:** Chronic liver disease, Liver cirrhosis, Nutrition, Sarcopenia, Survival, Malnutrition, Hepatology, Nutrition

## Abstract

This study aimed to evaluate whether the subjective global assessment (SGA) could effectively predict energy malnutrition, as assessed by indirect calorimetry, and mortality in hospitalized patients with cirrhosis. Energy malnutrition was defined by a nonprotein respiratory quotient (npRQ) < 0.85 using an indirect calorimetry. The usefulness of the SGA in identifying energy malnutrition and predicting mortality was assessed by the logistic regression and Cox proportional hazards models, respectively. Out of the 230 patients analyzed, 43% were found to have energy malnutrition. The distribution of SGA classifications was 54% for SGA-A, 32% for SGA-B, and 14% for SGA-C. Multivariable analysis indicated that both SGA-B (odds ratio, 3.59; 95% confidence interval [CI], 1.59–8.10) and SGA-C (odds ratio, 19.70; 95% CI, 3.46–112.00), along with free fatty acids (FFA), were independently linked to energy malnutrition. Regarding mortality, 125 patients (54%) died over a median follow-up period of 2.8 years. After adjustment, SGA-B (hazard ratio, 1.81; 95% CI, 1.08–3.03) and SGA-C (hazard ratio, 3.35; 95% CI, 1.28–8.76) were predictors of mortality in cirrhosis patients, while energy malnutrition and FFA were not. The SGA is a valuable tool for identifying energy malnutrition and predicting mortality in patients with cirrhosis.

**Supplementary Information:**

The online version contains supplementary material available at 10.1038/s41598-025-89803-6.

## Introduction

Malnutrition is prevalent among patients with cirrhosis and significantly affects key outcomes^1–6^. While less obvious in those with compensated cirrhosis, about half of the patients with decompensated cirrhosis suffer from malnutrition^3^. In cirrhosis, malnutrition often manifests as protein-energy malnutrition (PEM), stemming from deteriorating liver function, inadequate dietary intake, poor digestion and absorption of nutrients, and inefficient utilization of substrates^7,8^. The presence of PEM in patients with cirrhosis is linked to poorer disease outcomes, including diminished quality of life, increased cirrhosis complications, and higher mortality rates^7,8^. Protein malnutrition in cirrhosis patients is primarily due to decreased protein synthesis and elevated protein breakdown and is often assessed using serum albumin levels^7^. Energy malnutrition is characterized by changes in thermogenesis, such as reduced carbohydrate oxidation, heightened fat oxidation, and a consequent drop in nonprotein respiratory quotient (npRQ) as measured by indirect calorimetry^7^. Previous studies have demonstrated that an npRQ of 0.85 represents the threshold at which the predominant substrate for thermogenesis transitions from carbohydrates to fats^9^. Given its significant impact, clinical guidelines for liver disease nutrition recommend the use of indirect calorimetry in patients with cirrhosis^1–3,10^. In particular, the previous Japanese guidelines for cirrhosis recommended diagnosing energy malnutrition using npRQ < 0.85 to determine the need for initiating nutritional intervention^10^. However, its practical application in a daily clinical setting is limited by the associated costs, time demands, need for specialized equipment, and requirement for trained personnel. As a result, various biomarkers have been investigated as alternatives to npRQ. Using npRQ < 0.85 as a reference, a free fatty acid (FFA) level of 660 µEq/L was identified as an alternative marker for energy malnutrition^11^. In a similar study design, the assessment of liver functional reserves through the albumin–bilirubin (ALBI) score has been proposed as a marker for energy malnutrition^12^. However, the clinical relevance of these biomarkers to npRQ is relatively weak, and their availability is limited. Therefore, an alternative method to diagnose energy malnutrition and to initiate nutritional interventions are suggested as a future research question by the nutritional guideline^3^.

The subjective global assessment (SGA) is a nutritional evaluation tool that relies on data collected during a nutritional assessment^13^. Previous research has demonstrated that the SGA has excellent reproducibility and is linked to various clinical outcomes in patients with cirrhosis, including quality of life and mortality^3,14^. Consequently, nutritional guidelines endorse the SGA as a comprehensive method for diagnosing malnutrition in individuals with cirrhosis^1,3^. To bridge the gap between indirect calorimetry and contemporary approaches for assessing nutritional status, we hypothesize that the SGA may strongly correlate with energy malnutrition in cirrhosis patients. The ability of the SGA to diagnose malnutrition and to predict the outcomes has been well validated^15–17^. Yet, there is scant evidence on the relationship between the SGA and energy malnutrition in patients with cirrhosis. In addition, we hypothesized that the SGA is superior to other methods for evaluating energy malnutrition, such as npRQ and FFA, in predicting mortality in this longitudinal cohort study.

Given this context, the primary objective of our study is to explore the association between the SGA and energy malnutrition, defined by a npRQ < 0.85. The secondary goal is to compare the SGA and other energy malnutrition assessment techniques including npRQ and FFA for predicting mortality in patients with cirrhosis.

## Methods

### Study design and patients

This retrospective cohort study reviewed 345 Japanese hospitalized patients with cirrhosis who were admitted to Gifu University Hospital (Gifu, Japan), and received nutritional counseling from December 21, 2011, to April 18, 2014. The follow-up period extended until the last visit, death, or April 4, 2023, whichever came first. Due to the retrospective nature of the study, the need of informed consent was waived by the Institutional Review Board of the Graduate School of Medicine at Gifu University and the study protocol was reviewed and approved (approval number: 2023-087), ensuring compliance with the Declaration of Helsinki.

The study included patients with cirrhosis of any cause who were 18 years or older and underwent a nutritional assessment that encompassed the SGA and indirect calorimetry. Exclusion criteria were the presence of non-hepatic malignancies, acute liver failure, a history of liver or any other organ transplantation, life-threatening comorbidities (such as heart, respiratory, or renal failure), and patients who chose not to participate through the opt-out method.

### Outcomes and variables

The primary objective of this study was to identify predictors of energy malnutrition in patients with cirrhosis, as determined by a npRQ < 0.85 using indirect calorimetry. The secondary objective focused on identifying factors associated with mortality in these patients. Clinical variables, including biochemical parameters, Child–Pugh scores, the model for end-stage liver disease (MELD) scores, and ALBI grades, were evaluated within a week of the nutritional assessment. Body mass index (BMI) was calculated using the estimated dry weight, adjusted for fluid retention severity (5% for mild ascites, 10% for moderate ascites, 15% for severe ascites, and an additional 5% for bilateral pedal edema)^3^. Sarcopenia was diagnosed based on the second edition of the criteria proposed by the Japan Society of Hepatology, utilizing computed tomography imaging and handgrip strength tests^18^. Patients with both a reduced skeletal muscle mass index (< 42 cm^2^/m^2^ in males and < 38 cm^2^/m^2^in females) and handgrip strength (< 28 kg in males and < 18 kg in females) were diagnosed with sarcopenia^18^. Hepatocellular carcinoma and ascites were assessed through medical imaging, while hepatic encephalopathy was diagnosed using the West Haven criteria^19^.

### Assessment of energy malnutrition

Energy malnutrition was assessed using indirect calorimetry (Aeromonitor; Minato Medical Science, Osaka, Japan). Measurements were taken after the patients had fasted overnight for 12 h, just before breakfast, ensuring that they had been at rest for at least 30 min prior to the assessment. The indirect calorimeter measured oxygen consumption (VO_2_) and carbon dioxide production (VCO_2_) per minute. Total urine nitrogen (UN) was determined from a 24-hour urine sample. The npRQ was calculated using the formula: npRQ = (1.44 × VCO_2_ − 4.89 × UN) / (1.44 × VO_2_− 6.04 × UN)^20^. Patients with an npRQ < 0.85 were identified as having energy malnutrition^9^.

### Assessment of SGA

The SGA was conducted by experienced dietitians. It included an evaluation of five historical factors (changes in weight, dietary intake, gastrointestinal symptoms, functional capacity, and the impact of the disease on nutritional needs) and four physical examination findings (loss of subcutaneous fat, muscle wasting, ankle edema, sacral edema, and ascites). Based on these assessments, the patients were classified into three categories: A, indicating well-nourished; B, moderately malnourished; and C, severely malnourished^13^.

### Statistical methods

Continuous variables were presented as medians with interquartile ranges, while categorical variables were expressed in numbers and percentages. Differences between groups were analyzed using the Mann–Whitney *U*test for continuous variables and the chi-square test for categorical variables. The Bonferroni test was applied for post-hoc analyses across multiple groups. Logistic regression analysis identified predictors of an npRQ < 0.85, with results reported as odds ratios (ORs) and 95% confidence intervals (CIs). The model’s discriminative capability was assessed through sensitivity, specificity, positive predictive value (PPV), and negative predictive value (NPV). To evaluate mortality, the Cox proportional hazards model was utilized, and results were expressed as hazard ratios (HRs) with 95% CIs. Kaplan–Meier curves estimated mortality rates and group comparisons were made using the log-rank test. The covariate selection was predetermined by considering the multicollinearity of each variable^21^. All tests were two-sided, and a *p*-value < 0.05 was considered statistically significant. Statistical analyses were conducted using R (version 4.1.3, R Foundation for Statistical Computing, Vienna, Austria).

### Study size

To include approximately 10 variables in the multivariable analysis, we determined that 100 patients with energy malnutrition and mortality would be necessary. Given the estimated 50% prevalence of energy malnutrition and mortality in our cohort, we calculated that a total of 200 patients would be required for the analysis.

## Results

### Baseline characteristics of patients with cirrhosis

Among the 345 patients assessed for eligibility, 115 were excluded owing to missing data (*n* = 111), acute liver failure (*n* = 2), and lung cancer (*n* = 2), leaving 230 patients for analysis. Of these, 68% were males, with a median age of 71 years and a BMI of 22 kg/m^2^. The primary causes of cirrhosis included hepatitis C (61%), followed by alcohol-related causes (16%), other causes (15%), and hepatitis B (7%). Hepatocellular carcinoma and sarcopenia were observed in 66% and 20% of the patients, respectively. The median scores for Child–Pugh, MELD, and ALBI were 6, 8, and − 2.03, respectively. Regarding the SGA, 54% were classified as well-nourished (SGA-A), 32% as moderately nourished (SGA-B), and 14% as severely malnourished (SGA-C). The median npRQ was 0.84, with 43% of patients experiencing energy malnutrition (npRQ < 0.85) (Table 1).

The comparison between patients with and without energy malnutrition revealed significant differences. Those with energy malnutrition had worse SGA than those without energy malnutrition (SGA-A: 39% vs. 73%; SGA-B: 39% vs. 23%; SGA-C: 23% vs. 4%; *p* < 0.001). Additionally, patients with energy malnutrition exhibited significantly worse Child–Pugh, MELD, and ALBI scores. They also had higher serum bilirubin and FFA levels, along with lower serum albumin levels and branched-chain amino acids to tyrosine ratio (Table 1).

**Table 1:** Baseline characteristics of patients with cirrhosis, divided by energy malnutrition.

	Total cohort	npRQ ≥ 0.85	npRQ < 0.85	*p*-value*
Characteristic	(*n* = 230)	(*n* = 129)	(*n* = 100)	
Age, years	71 (63–78)	71 (64–77)	71 (62–79)	0.801
Male	157 (68)	70 (69.3)	87 (67.4)	0.874
Body mass index, kg/m²	22 (20–24)	22 (20–24)	22 (19–24)	0.811
Etiology of cirrhosis				
Hepatitis B virus	18 (7)	8 (8)	10 (8)	0.482
Hepatitis C virus	141 (61)	66 (65)	75 (58)	
Alcohol-related	37 (16)	12 (12)	25 (19)	
Others	34 (15)	15 (15)	19 (15)	
SGA				< 0.001
SGA-A	124 (54)	74 (73)	50 (39)	
SGA-B	73 (32)	23 (23)	50 (39)	
SGA-C	33 (14)	4 (4)	29 (23)	
Hepatocellular carcinoma	152 (66)	64 (63)	88 (68)	0.528
Sarcopenia	47 (20)	19 (19)	28 (22)	0.707
Skeletal muscle mass index (kg/m^2^)	43 (39–50)	43 (39–49)	44 (39–50)	0.834
Handgrip strength (kg)	25 (19–32)	27 (21–33)	23 (18–31)	0.017
npRQ	0.84 (0.78–0.90)	0.91 (0.88–0.95)	0.79 (0.76–0.82)	< 0.001
Child-Pugh score	6 (5–8)	7 (6–9)	9 (7–11)	< 0.001
MELD score	8 (7–910	5 (5–7)	6 (5–9)	0.001
ALBI score	−2.03 (−2.38–−1.50)	−2.22 (−2.52–−1.84)	−1.85 (−2.31–−1.27)	< 0.001
Albumin, g/dL	3.4 (2.9–3.7)	3.5 (3.1–3.8)	3.2 (2.5–3.6)	< 0.001
Creatinine, mg/dL	0.74 (0.61–0.88)	0.75 (0.61–0.86)	0.73 (0.61–0.91)	0.796
Sodium, mEq/L	139 (137–140)	139 (137–140)	138 (136–140)	0.232
Total bilirubin, mg/dL	1.0 (0.7–1.6)	0.90 (0.70–1.30)	1.10 (0.80–1.90)	0.001
International normalized ratio	1.05 (0.99–1.14)	1.04 (0.98–1.11)	1.06 (1.00–1.16)	0.065
Ammonia, mg/dL	54 (42–78)	52 (42–70)	58 (41–80)	0.468
Zinc, µg/dL	77 (67–93)	84 (71–95)	74 (62–88)	0.001
BTR	4.20 (3.19–5.28)	4.40 (3.35–5.33)	3.86 (2.92–5.20)	0.088
Free fatty acid, µEq/L	687 (510–879)	573 (454–725)	757 (582–918)	< 0.001

Values are presented as numbers (percentages) or medians (interquartile ranges).

*Clinical characteristics between the two groups were compared using the Chi-square test or Mann–Whitney *U* test. Abbreviations: ALBI, albumin–bilirubin; BTR, branched-chain amino acid-to-tyrosine ratio; MELD, model for end-stage liver disease; npRQ, nonprotein respiratory quotient; SGA, subjective global assessment

## Discussion

This study evaluated various nutritional assessment methods for identifying energy malnutrition in cirrhosis patients. A notable discovery was that the SGA is effective for detecting energy malnutrition in this patient group. Another significant finding is the strong correlation between SGA scores and mortality rates among cirrhosis patients. These insights bridge the gap between the “impractical” and “practical” nutritional assessment methods, paving the way for a sound strategy for nutritional assessment and intervention to enhance patient outcomes in cirrhosis.

Identifying energy malnutrition in cirrhosis patients continues to be a challenge. Previous research indicated that serum FFA levels might serve as an alternative to the npRQ because of a moderate correlation (*r* = −0.39).^11^Consequently, earlier guidelines recommended nutritional interventions for patients with FFA levels above 660 µEq/L as an alternate way to gauge energy malnutrition^10^. However, FFA testing is not widely used in routine practice, and Japanese health insurance does not cover FFA testing for cirrhosis patients. As a result, recent guidelines no longer recommend relying on npRQ and FFA, instead suggesting the use of BMI, liver functional reserves, sarcopenia, and nutritional assessment tools such as SGA to identify malnutrition in patient with cirrhosis^3,5,6^.

Our study determined that the SGA is an effective indicator for identifying energy malnutrition in cirrhosis patients. Moreover, multivariable analysis revealed that SGA is more closely associated with the npRQ than previously reported markers such as the Child–Pugh and ALBI scores, the branched-chain amino acid-to-tyrosine ratio, and serum zinc levels^12,22–24^. In terms of discriminative ability, SGA categories B and C displayed similar sensitivity and higher specificity compared to a FFA > 660 µEq/L, leading to higher PPV and NPV for SGA than those observed for FFA. The strong link between SGA and energy malnutrition can be attributed to the components evaluated by the SGA. Factors like dietary intake and weight changes, which are integral to the SGA, are well-established causes and manifestations of PEM^7,8^. Furthermore, the SGA’s assessment of ascites and edema reflects liver functional reserves, which may be the consequence of PEM^7,8^. Given these insights, the SGA not only captures disease-specific aspects of malnutrition in patients with cirrhosis but also serves as a reliable method for pinpointing energy malnutrition and guiding nutritional interventions.

Another significant finding from our study was that the SGA proved useful for stratifying mortality risk in cirrhosis patients. While several studies have explored the role of malnutrition, as assessed by the SGA, in this patient group, the impact of SGA on outcomes remains a subject of debate, highlighting the need for further research. Previous investigations have suggested that malnutrition, as determined by SGA, had a lesser ability to predict mortality than muscle mass and handgrip strength in cirrhosis patients^25,26^. Conversely, another study indicated that both SGA and handgrip strength independently predicted outcomes post-liver transplantation^27^. In our research, SGA’s influence on mortality was found to be independent of liver functional reserves and more significant than that of sarcopenia, as assessed by muscle mass and handgrip strength. Moreover, SGA was able to stratify mortality risk in cirrhosis patients, whereas other indicators of energy malnutrition, such as the npRQ and FFA, did not achieve statistical significance in multivariable models. This aligns with the results of recent studies that have identified SGA as an independent predictor of mortality in cirrhosis patients, regardless of liver functional reserves^14 ,28^. Despite evidence showing that energy malnutrition, assessed via indirect calorimetry, has a substantial impact on mortality in cirrhosis patients, our findings endorse the use of SGA as a standard method for mortality risk stratification in this demographic.

Our study is not without limitations. First, being a single-center retrospective study, it may contain biases and confounders. Second, with viral hepatitis being the predominant cause of cirrhosis among our subjects, our results may not extend to other etiologies. Third, certain established nutritional assessment tools, such as the Royal Free Hospital-global assessment, were not evaluated in this study^29^. Thus, further research encompassing multiple centers and various nutritional assessment tools is needed to validate the effectiveness of SGA in predicting energy malnutrition and mortality in patients with cirrhosis.

In conclusion, this study has demonstrated that the SGA is an effective nutritional tool for identifying energy malnutrition in patients with cirrhosis. Furthermore, malnutrition diagnosed with SGA can stratify mortality risk more effectively than energy malnutrition alone. Our findings contribute to the development of strategies for nutritional assessment and intervention aimed at improving outcomes for patients with cirrhosis.

### Impact of the SGA on energy malnutrition in patients with cirrhosis

**Fig. 1:**
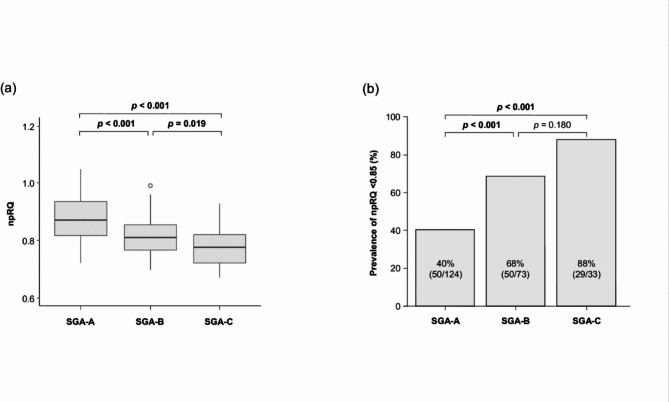
(a) Boxplots of npRQ and (b) prevalence of energy malnutrition (npRQ < 0.85) in patients with cirrhosis divided by SGA. The Bonferroni test was used for multiple pairwise comparisons. Abbreviations: npRQ, nonprotein respiratory quotient; SGA, subjective global assessment.

Among the cirrhosis patients studied, 100 (43%) were diagnosed with energy malnutrition, as indicated by a npRQ < 0.85. In multivariable analysis, Model 1, which included the Child-Pugh score, revealed that SGA-B (OR, 3.59; 95% CI, 1.59–8.10; *p* = 0.002), SGA-C (OR, 19.70; 95% CI, 3.46–122.00; *p* < 0.001), and FFA were independently associated with energy malnutrition. Similar findings were observed in Model 2, which incorporated the MELD score, and Model 3, which included the ALBI score (Table 2). The details of the univariate analysis are presented in Supplementary Table S1. The median npRQ values and the prevalence of energy malnutrition (npRQ < 0.85) in patients classified as SGA-A, SGA-B, and SGA-C were 0.87, 0.81, and 0.78, respectively (Fig. 1a), and 40%, 68%, and 88% (Fig. 1b), respectively.

**Table 2:** Factors associated with energy malnutrition in patients with cirrhosis.

	Model 1		Model 2		Model 3	
Characteristic	OR (95% CI)	*p*-value*	OR (95% CI)	*p*-value*	OR (95% CI)	*p*-value*
Age, years	0.99 (0.97–1.02)	0.607	1.00 (0.97–1.02)	0.721	0.99 (0.97–1.02)	0.675
Male	0.94 (0.50–1.76)	0.842	0.95 (0.51–1.79)	0.876	0.95 (0.51–1.79)	0.883
Body mass index, kg/m²	1.09 (0.99–1.19)	0.077	1.05 (0.96–1.15)	0.256	1.05 (0.96–1.15)	0.284
SGA						
SGA-A^†^	1		1		1	
SGA-B	3.59 (1.59–8.10)	0.002	2.54 (1.23–5.23)	0.012	2.49 (1.18–5.21)	0.016
SGA-C	19.70 (3.46–112.00	< 0.001	7.46 (1.70–32.70)	0.007	7.39 (1.79–30.40)	0.006
Sarcopenia	0.97 (0.44–2.15)	0.935	0.98 (0.44–2.18)	0.966	0.97 (0.44–2.13)	0.937
Child-Pugh score	0.81 (0.62–1.07)	0.137				
MELD score			1.02 (0.89–1.18)	0.78		
ALBI score					1.15 (0.57–2.32)	0.706
Zinc, mg/dL	1.00 (0.98–1.01)	0.593	1.00 (0.98–1.02)	0.958	1.00 (0.98–1.02)	0.977
Free fatty acid, mEq/L	1.00 (1.00–1.00)	0.008	1.00 (1.00–1.00)	0.011	1.00 (1.00–1.00)	0.009

*Analysis was performed using the logistic regression model.

†Reference group.

Abbreviations: ALBI, albumin–bilirubin; CI, confidence interval; MELD, model for end-stage liver disease; OR, odds ratio; SGA, subjective global assessment

### Ability of SGA to identify energy malnutrition in patients with cirrhosis

Based on the results from multivariable analyses, the discriminative capacity of the SGA and FFA to identify energy malnutrition was evaluated. For SGA categories B and C, the sensitivity, specificity, PPV, and NPV were 0.61, 0.73, 0.75, and 0.58, respectively. When specifically considering the cutoff for SGA-C, these values were 0.23, 0.96, 0.88, and 0.49, respectively. Using the recommended cutoff value of FFA > 660 µEq/L,^10,11^ the sensitivity, specificity, PPV, and NPV were 0.64, 0.60, 0.67, and 0.57, respectively.

### Impact of SGA on mortality in patients with cirrhosis

**Fig. 2:**
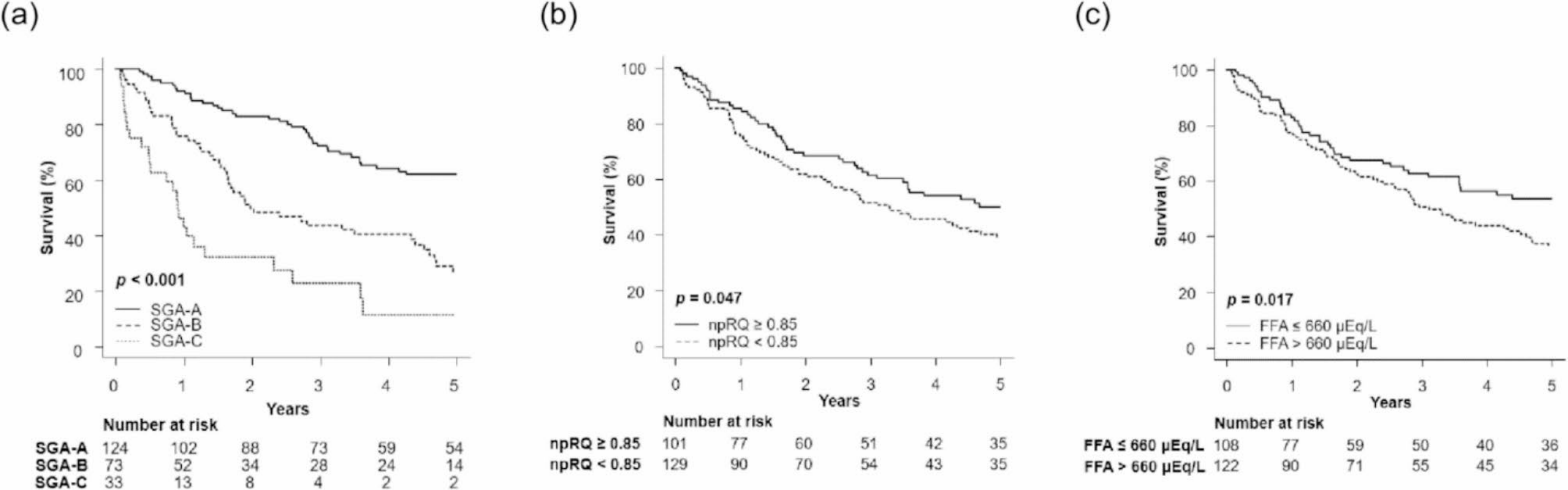
Overall survival of patients with cirrhosis according to (a) SGA, (b) npRQ, and (c) FFA. The survival curve was estimated using the Kaplan–Meier method, and the differences between groups were compared using the log-rank test. Abbreviations: FFA, free fatty acid; npRQ, nonprotein respiratory quotient; SGA, subjective global assessment.

During a median follow-up period of 2.8 years (interquartile range, 0.9–6.3 years), 125 (54%) patients died. The leading causes of death were hepatocellular carcinoma (62%), liver failure (24%), and other causes (14%). The 5-year survival rates were 62%, 27%, and 11% in patients with SGA-A, SGA-B, and SGA-C (*p* < 0.001; Fig. 2a), 50% and 39% in patients with and without energy malnutrition (*p* = 0.047; Fig. 2b), 53%, and 37% in patients with FFA ≤ 660 µEq/L and > 660 µEq/L, respectively (*p* = 0.017; Fig. 2c).

Multivariable analysis Model 1, which included the Child-Pugh score, indicated that SGA-B (HR, 1.81; 95% CI, 1.08–3.03; *p* = 0.025) and SGA-C (HR, 3.35; 95% CI, 1.28–8.76; *p* = 0.014) were associated with mortality in patients with cirrhosis, whereas energy malnutrition and FFA were not. Similar outcomes were observed in models incorporating the MELD score and the ALBI score (Table 3). Details of the univariate analysis are presented in Supplementary Table S2.

**Table 3:** Factors associated with mortality in patients with cirrhosis.

	Model 1		Model 2		Model 3	
Characteristic	HR (95% CI)	*p*-value*	HR (95% CI)	*p*-value*	HR (95% CI)	*p*-value*
Age, years	1.02 (0.99–1.04)	0.061	1.02 (0.99–1.04)	0.181	1.01 (0.99–1.03)	0.295
Male	1.21 (0.79–1.85)	0.391	1.16 (0.75–1.78)	0.51	1.14 (0.73–1.76)	0.565
Body mass index, kg/m²	0.94 (0.88–1.00)	0.05	0.96 (0.91–1.02)	0.206	0.96 (0.90–1.02)	0.138
SGA						
SGA-A^†^	1		1		1	
SGA-B	1.81 (1.08–3.03)	0.025	2.36 (1.43–3.90)	< 0.001	2.17 (1.31–3.60)	0.003
SGA-C	3.35 (1.28–8.76)	0.014	7.98 (3.36–18.94)	< 0.001	7.04 (3.02–16.42)	< 0.001
Hepatocellular carcinoma	5.12 (2.91–8.99)	< 0.001	5.13 (2.88–9.12)	< 0.001	4.96 (2.80–8.81)	< 0.001
npRQ < 0.85	1.06 (0.69–1.63)	0.798	0.94 (0.61–1.44)	0.761	0.94 (0.61–1.44)	0.77
Sarcopenia	0.59 (0.35–0.99)	0.044	0.70 (0.42–1.17)	0.171	0.98 (0.44–2.18)	0.966
Child-Pugh score	1.39 (1.17–1.64)	< 0.001				
MELD score			1.06 (0.98–1.15)	0.148		
ALBI score					1.77 (1.05–2.99)	0.032
Sodium, mEq/L	0.92 (0.87–0.98)	0.011	0.92 (0.86–0.98)	0.01	0.91 (0.86–0.97)	0.004
Zinc, mg/dL	1.01 (1.00–1.02)	0.065	1.01 (0.99–1.02)	0.352	1.01 (1.00–1.02)	0.115
BTR	0.99 (0.86–1.13)	0.846	0.93 (0.81–1.07)	0.29	0.95 (0.82–1.09)	0.449
Free fatty acid, mEq/L	1.00 (1.00–1.00)	0.415	1.00 (1.00–1.00)	0.847	1.00 (1.00–1.00)	0.667

*Analysis was performed using the Cox proportional hazards model.

^†^Reference group.

Abbreviations: ALBI, albumin–bilirubin; CI, confidence interval; HR, hazard ratio; MELD, model for end-stage liver disease; npRQ, nonprotein respiratory quotient; SGA, subjective global assessment.

## Electronic supplementary material

Below is the link to the electronic supplementary material.

Supplementary Material 1

## Data Availability

The datasets generated and/or analyzed data during the current study are available from the corresponding author upon reasonable request.
