# Supplementary material for: Nutritional assessment using subjective global assessment identifies energy malnutrition and predicts mortality in patients with liver cirrhosis
Source: Sci Rep. 2025 Feb 9;15:4831. doi: 10.1038/s41598-025-89803-6 (PMC11808070; doi:10.1038/s41598-025-89803-6)
Supplement: Supplementary file 1 — Supplementary Material 1 [file 41598_2025_89803_MOESM1_ESM.docx]

**Supplementary Information**

**Title: Nutritional assessment using subjective global assessment identifies energy malnutrition and predicts mortality in patients with liver cirrhosis**

Takao Miwa^1^, Tatsunori Hanai^1,2^, Kayoko Nishimura^2^, Sachiyo Hirata^2^, Shinji Unome^1^, Yuki Nakahata^1,3^, Kenji Imai^1^, Atsushi Suetsugu^1^, Koji Takai^1,4^, Masahito Shimizu^1^

1. Department of Gastroenterology/Internal Medicine, Graduate School of Medicine, Gifu University, Gifu, Japan
2. Center for Nutrition Support & Infection Control, Gifu University Hospital, Gifu, Japan
3. Department of Gastroenterology, Asahi University Hospital, Gifu, Japan
4. Division for Regional Cancer Control, Graduate School of Medicine, Gifu University, Gifu, Japan

**Supplementary Table S1.** Univariate analysis of factors associated with energy malnutrition in patients with cirrhosis

| Characteristic | OR (95% CI) | *p*-value* |
| --- | --- | --- |
| Age, years | 0.99 (0.97–1.02) | 0.632 |
| Male | 0.92 (0.52–1.61) | 0.763 |
| Body mass index, kg/m² | 1.00 (0.93–1.08) | 0.967 |
| Etiology of cirrhosis |  |  |
| Hepatitis B virus^†^ | 1.00 |  |
| Hepatitis C virus | 0.91 (0.34–2.44) | 0.850 |
| Alcohol-related | 0.67 (0.52–5.30) | 0.387 |
| Others | 1.01 (0.32–3.20) | 0.982 |
| SGA |  |  |
| SGA-A^†^ | 1.00 |  |
| SGA-B | 3.22 (1.75–5.92) | <0.001 |
| SGA-C | 10.73 (3.55–32.40) | <0.001 |
| Hepatocellular carcinoma | 1.24 (0.72–2.15) | 0.441 |
| Sarcopenia | 1.20 (0.62–2.29) | 0.589 |
| Skeletal muscle mass index (kg/m^2^) | 1.00 (0.97–1.03) | 0.810 |
| Handgrip strength (kg) | 0.96 (0.93–0.99) | 0.013 |
| Child-Pugh score | 1.29 (1.11–1.50) | <0.001 |
| MELD score | 1.21 (1.09–1.35) | <0.001 |
| ALBI score | 2.61 (1.66–4.11) | <0.001 |
| Albumin, g/dL | 0.41 (0.26–0.64) | <0.001 |
| Creatinine, mg/dL | 1.57 (0.62–4.01) | 0.331 |
| Sodium, mEq/L | 0.93 (0.85–1.02) | 0.141 |
| Total bilirubin, mg/dL | 1.87 (1.28–2.74) | 0.001 |
| International normalized ratio | 8.18 (1.24–54.02) | 0.029 |
| Ammonia, mg/dL | 1.00 (1.00–1.01) | 0.375 |
| Zinc, μg/dL | 0.99 (0.97–1.00) | 0.021 |
| BTR | 0.90 (0.76–1.07) | 0.219 |
| Free fatty acid, μEq/L | 1.00 (1.00–1.00) | <0.001 |

*Analysis was performed using the logistic regression model.

^†^Reference group

Abbreviations: ALBI, albumin–bilirubin; BTR, branched-chain amino acid-to-tyrosine ratio; CI, confidence interval; MELD, model for end-stage liver disease; OR, odds ratio; SGA, subjective global assessment

**Supplementary Table S2.** Univariate analysis of factors associated with mortality in patients with cirrhosis

| Characteristic | HR (95% CI) | *p*-value* |
| --- | --- | --- |
| Age, years | 1.03 (1.01–1.05) | 0.002 |
| Male | 1.06 (0.72–1.54) | 0.781 |
| Body mass index, kg/m² | 0.93 (0.88–0.98) | 0.004 |
| Etiology of cirrhosis |  |  |
| Hepatitis B virus^†^ | 1.00 |  |
| Hepatitis C virus | 1.08 (0.57–2.03) | 0.810 |
| Alcohol-related | 1.29 (0.62–2.66) | 0.495 |
| Others | 0.71 (0.32–1.57) | 0.399 |
| SGA |  |  |
| SGA-A^†^ | 1.00 |  |
| SGA-B | 2.31 (1.55–3.43) | <0.001 |
| SGA-C | 5.31 (3.26–8.63) | <0.001 |
| Hepatocellular carcinoma | 2.92 (1.88–4.54) | <0.001 |
| Sarcopenia | 0.99 (0.63–1.54) | 0.949 |
| Skeletal muscle mass index (kg/m^2^) | 0.98 (0.96–1.00) | 0.043 |
| Handgrip strength (kg) | 0.98 (0.96–1.00) | 0.023 |
| npRQ <0.85 | 1.44 (1.00–2.06) | 0.049 |
| Child-Pugh score | 1.33 (1.23–1.44) | <0.001 |
| MELD score | 1.17 (1.11–1.22) | <0.001 |
| ALBI score | 2.34 (1.81–2.99) | <0.001 |
| Albumin, g/dL | 0.43 (0.34–0.56) | <0.001 |
| Creatinine, mg/dL | 1.94 (1.08–3.12) | 0.029 |
| Sodium, mEq/L | 0.88 (0.83–0.93) | <0.001 |
| Total bilirubin, mg/dL | 1.44 (1.27–1.60) | <0.001 |
| International normalized ratio | 11.51 (3.90–31.71) | <0.001 |
| Ammonia, mg/dL | 1.00 (1.00–1.01) | 0.105 |
| Zinc, μg/dL | 0.98 (0.97–0.99) | <0.001 |
| BTR | 0.79 (0.69–0.89) | <0.001 |
| Free fatty acid, μEq/L | 1.00 (1.00–1.00) | 0.016 |

*Analysis was performed using the Cox proportional hazards model.

^†^Reference group

Abbreviations: ALBI, albumin–bilirubin; BTR, branched-chain amino acid-to-tyrosine ratio; CI, confidence interval; HR, hazard ratio; MELD, model for end-stage liver disease; npRQ, nonprotein respiratory quotient; SGA, subjective global assessment
